# Management of aplastic anemia in a woman during pregnancy: a case report

**DOI:** 10.1186/1752-1947-5-66

**Published:** 2011-02-15

**Authors:** Krista JM Stibbe, Hajo IJ Wildschut, Pieternella J Lugtenburg

**Affiliations:** 1 Department of Obstetrics of Gynaecology, Erasmus University Medical Center, PO Box 2040, 3000 CA Rotterdam, The Netherlands; 2 Department of Obstetrics of Gynaecology, Erasmus University Medical Center, PO Box 2040, 3000 CA Rotterdam, The Netherlands; 3 Department of Hematology, Erasmus University Medical Center, PO Box 2040, 3000 CA Rotterdam, The Netherlands

## Abstract

**Introduction:**

Aplastic anemia is a rare disease caused by destruction of pluripotent stem cells in bone marrow. During pregnancy it could be life-threatening for both mother and child. The only causal therapy for aplastic anemia is bone marrow transplantation, which is contraindicated during pregnancy because of potential embryo toxicity. Treatment options are erythrocytes and platelet transfusions and immunosuppressive therapy. There is, however, no agreement about the optimal supportive care and treatment regime for this disorder during pregnancy.

**Case Presentation:**

A 26-year-old nulliparous Asian woman with an uneventful medical history was admitted to the hospital at 14 weeks' gestation because of excessive vomiting. Routine laboratory tests showed pancytopenia (Hb 3.5 mmol/L, leukocytes 3.5 *10^9^/L, platelets 45 *10^9^L). A bone marrow biopsy confirmed aplastic anemia. Methylprednisolon, cyclosporine A, packed cells and platelet transfusions were initiated. At 33 weeks she developed neutropenia (0.1 *10^9^/L) for which oral colistin and tobramycin were given prophylactically. At 35 weeks labor was induced, during which she developed a fever of 38.2°C. She gave birth spontaneously to a healthy son weighing 2415 grams, who had no signs of pancytopenia. After delivery the blood count of the patient did not recover and did not respond to medication. Eighteen weeks after delivery she died of sepsis complicated by cerebral bleeding and infarction due to severe thrombocytopenia and neutropenia, despite optimal supportive treatment.

**Conclusion:**

This potential life-threatening disease has a relatively good prognosis for both mother and child after optimal treatment. Transfusion during pregnancy is the first choice treatment with recommended hemoglobin levels of >5.5 mmol/L and platelet counts of >20 *10^9^/L. Cyclosporine A seems a reasonable alternative therapy with a reported success rate in non-pregnant patients of 70% when combined with antithymocyte globuline. Our patient died 18 weeks postpartum from cerebral bleeding and infarction due to severe thrombocytopenia despite intensive supportive treatment, methylprednisolon and cyclosporine A.

## Introduction

Aplastic anemia is a rare disease caused by destruction of pluripotent stem cells in bone marrow with an annual incidence of 2 to 6/1.000.000 [1]. In contrast to the term 'aplastic anemia', suggesting suppression of erythropoetic cell lines, all cell lines may be affected [2]. Depending on affected cell lines, aplastic anemia is associated with fatigue, bleeding due to thrombocytopenia and recurrent infections due to neutropenia [3]. The diagnosis 'aplastic anemia' is confirmed by hypocellularity of the bone marrow. The remaining cells are morphologically unaffected without malignant infiltration. Potential triggers for the onset of aplastic anemia include T-cell mediated auto-immune disease, iatrogenic agents, viral infection and pregnancy [1]. There is, however, no causal relation between pregnancy and the onset of aplastic anemia [4]. This notion is supported by the similar incidence of aplastic anemia in men and women [1]. During pregnancy bone marrow transplantation is contraindicated because of potential embryo toxicity [5]. There are no clear guidelines for the management of aplastic anemia during pregnancy. Is immunosuppressive treatment more effective than supportive therapy consisting of erythrocytes and platelet transfusion and antibiotics?

## Case presentation

**Figure 1:**
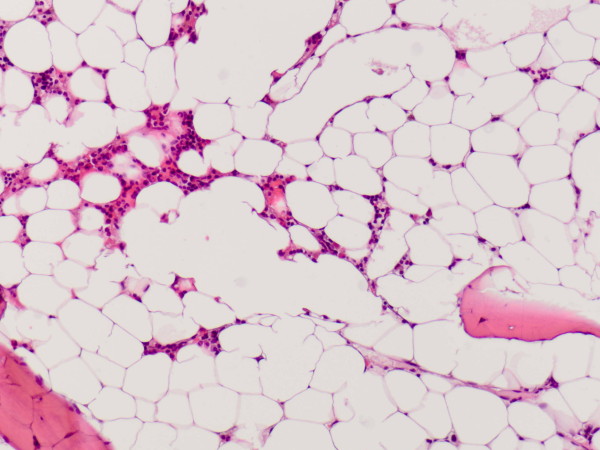
**Hypocellular bone marrow showed only a little hematopoesis and many fat cells**.

A 26-year-old nulliparous Asian woman with an uneventful medical history was admitted to a district hospital at 14 weeks' gestation because of excessive vomiting. Laboratory tests showed pancytopenia (Hb 3.5 mmol/l, Ht 0.17, MCV 87, reticulocytes 28.6 *10^9^/l, leukocytes 3.5 *10^9^/L with differentiation 41% neutrophiles, 50% lymphocytes and 8% monocytes, platelets 45 *10^9^L). No iron, folic acid and vitamin B12 depletion were encountered. She did not use medications and had not been exposed to toxic materials. A bone marrow biopsy was taken showing poor cellular bone marrow without signs of malignancy. Aplastic anemia was the most likely diagnosis. The patient was transferred to our academic hospital for further diagnostic evaluation, counseling and treatment. Repeated bone marrow tests, including immunophenotyping and cytogenetic tests, did not show abnormal cell populations. Bone marrow biopsy showed hypocellular marrow with a percentage less than 5% (Figure 1) without specific markers for other causes of pancytopenia, such as acute leukemia, myelodysplastic syndrome, hairy cell leukemia, lymphoma, myelofibrosis or anorexia nervosa. In concordance with the diagnosis of aplastic anemia a small population of paroxysmal nocturnal hemoglobinuria (PNH) positive cells was detected in peripheral blood.

**Figure 2:**
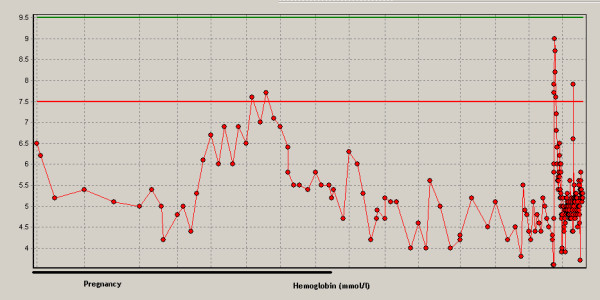
**Hemoglobin (mmol/l) during pregnancy to the moment of death**. Normal ranges are between the lines.

**Figure 3:**
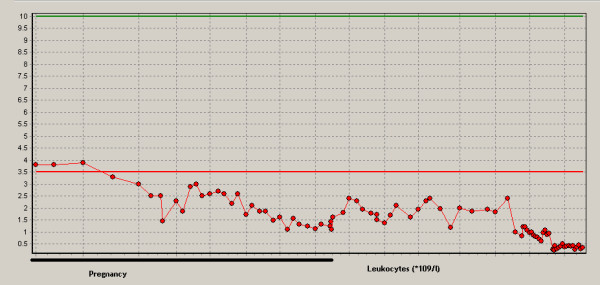
Course of leukocyte numbers (*10^9^/l).

**Figure 4:**
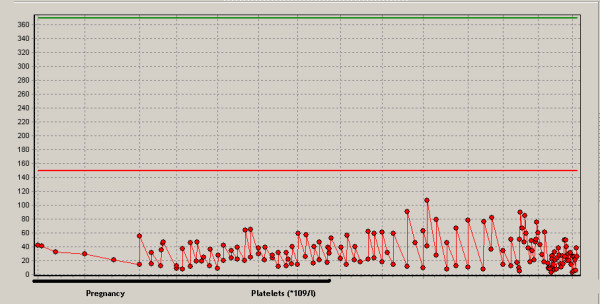
Course of platelet numbers (*10^9^/l).

Aplastic anemia was assumed to originate during pregnancy because six months before admittance to the hospital our patient's hemoglobin level was normal (8.1 mmol/l; normal range: 7.5 to 10.0 mmol/l). During hospital admission the disease progressed resulting in erythrocyte (270 mL dosage consisting of Ht 0.57 l/l) and platelet transfusion (310 mL consisting of 340 *10^9 ^platelets) with target platelet count 10 *10^9^/l twice a week. Methylprednisolon was started together with cyclosporine A (CsA) in a dosage of 5 mg/kg twice a day (bd). The patient did not respond (Figures 2, 3, 4). Moreover, she developed HLA-antibodies against platelets, complicating the search for suitable platelet donors. At 35 weeks the patient became neutropenic, for which oral colistin and tobramycin were given prophylactically.

**Figure 5:**
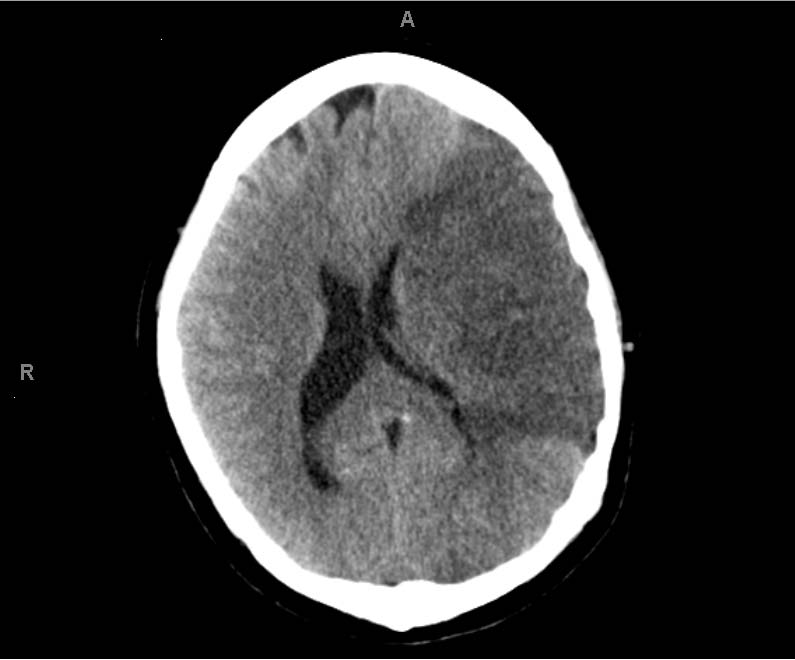
**CT scan cerebrum performed on the last day of the patient's life**.

At 35 weeks labor was induced electively for logistic reasons. Sufficient platelet donors with HLA identical platelet transfusions were guaranteed. During delivery the patient had a fever (38.2°C). The fetus responded with tachycardia. Since intra uterine infection was suspected, amoxicillin and clavulanic acid were started intravenously. She gave birth spontaneously to a healthy son weighing 2415 grams with a five-minute Apgar score of 10. Estimated blood loss during delivery was 100 ml. After delivery severe pancytopenia with neutrophile number <0.05 *10^9^/l persisted. She remained erythrocyte and platelet transfusion-dependent despite CsA treatment. After delivery laboratory tests still showed severe pancytopenia despite transfusions (Hb 5.4 mmol/l, platelets 53 *10^9^/l, leukocytes 1.5 *10^9^/l and neutrophils <0.05 *10^9^/l). Treatment with CsA was continued without success. Therefore, ATG was planned to be added three months after delivery. However, during hospital admission the patient had a fever of 41.2°C and gingival bleeding. No microbiological origin was found. The patient was treated with imipenem and ATG treatment was postponed. The next day the patient had diarrhea, headache and a maculopapular rash, probably due to medication use. Therefore, all medications were stopped for three days. She was discharged from the hospital to recover from the rash and fever. However, two days later she returned to the hospital because of severe vomiting, diarrhea and headache. She became hypotensive and hypoxic needing admission to the intensive care unit. Because of *Staphylococcus aureus *bacteremia, antibiotic treatment was started (meropenem and vancomycin). She needed artificial respiration because of progressive dyspnea. She developed bilateral pneumonia followed by a pneumothorax in the left lung needing a thoracal drain. Antibiotic treatment was switched to flucloxacillin. Two days later she had an anisocoria, that is, unequal pupil sizes and Glasgow coma score of E1M4(left)Vt. Platelets were 26 *10^9^/l (target platelet count 20 *10^9^/l). The previous day platelets had been 3 *10^9^/l although HLA matched platelet transfusion five times a day. A computed tomography scan of the cerebrum showed a total left arteria cerebri media infarction with bleeding, compression of the left lateral ventricle and midline shift to the right (Figure 5).

Because of the poor prognosis, based on the combination of very severe aplastic anemia and cerebral infarction, further treatment was stopped and the patient died 18 weeks after delivery. At present the child is healthy.

## Discussion

Little research has been published about therapy for aplastic anemia during pregnancy. In fact, only case reports and series with small sample sizes are available. In young non-pregnant patients first choice therapy for aplastic anemia is allogenic stem cell transplantation with a five-year survival of 70 to 80% [1]. However, stem cell transplantation is not feasible during pregnancy because of the teratogenic effects of the immunotherapy and radiotherapy for the unborn child [5]. Pregnancy termination to start bone marrow transplantation was not recommended because of the relatively good prognosis for both mother and child. During pregnancy supportive therapy with erythrocyte and platelet transfusions is a widely used, reasonable alternative. As described in this case, the benefit of transfusions to prevent bleeding should be weighed against the likelihood of developing HLA antibodies and hemochromatosis in the mother [6]. Therefore, we started with a low number and frequency of platelets transfused and increased the frequency after persistent thrombocytopenia [7]. In case the patient responded insufficiently to supportive therapy, immunotherapy with ATG, CsA and/or methylprednisolon could be started. These therapies are used regularly in non-pregnant patients with a hematological response of 40 to 70% [1]. Lesser experience has been gained with ATG treatment during pregnancy, only two cases out of 75. Moreover, both patients died [2,8]. Outside of pregnancy CsA had comparable results with ATG in a randomized controlled multicenter study [9]. According to this study the hematological responses for CsA and ATG after 12 months were 31.6% and 30% respectively. The one year survival for CsA and ATG was 64 to 70% [9].

Several case reports refer to Knispel *et al*. describing a maternal mortality of 20 to 60%. However, the mortality rate in that article published in 1976 differs from the currently reported mortality of 2.7%, probably due to better immunosuppressive treatment and supportive care [2-6,8,10,11]. Unfortunately our pregnant patient with very severe aplastic anemia died after intensive supportive and immunosuppressive treatment with methylprednisolon and CsA during and after pregnancy. She was treated according to the best available treatment based on the literature. She was not treated with ATG during pregnancy because of the bad responses of the two described patients in the literature. Because of the reasonable clinical condition of the patient and good condition of the fetus, there was no indication to terminate the pregnancy early.

## Conclusion

Aplastic anemia is a serious condition which may manifest during pregnancy. The seriousness depends on the degree of bone marrow suppression. Most pregnant patients will have full-term pregnancies with a healthy child. Fortunately, aplastic anemia has a low maternal mortality due to treatment. During severe aplastic anemia or complications caused by the supportive therapy (erythrocyte and platelet transfusions and antibiotics) ATG, methylprednisolon and/or CsA could be started. Nonetheless, our patient died 18 weeks postpartum from sepsis, cerebral bleeding and infarction due to severe thrombocytopenia despite intensive supportive treatment, methylprednisolon and CsA. This case shows that aplastic anemia during pregnancy is potentially a life-threatening condition despite the favorable prognosis for both mother and child.

## Abbreviations

ATG: antithymocyte globulin; CsA: Cyclosporin A; Hb: hemoglobin; HLA: human leukocyte antigen; Ht: hematocrit; MCV: mean corpuscular volume; PNH: paroxysmal nocturnal hemoglobinuria.

## Consent

Written informed consent was obtained from the patient and her parents for publication of this case report and accompanying images. A copy of the written consent is available for review by the Editor-in-Chief of this journal.

## Competing interests

The authors declare that they have no competing interests.

## Authors' contributions

HW provided antenatal care to the patient during the pregnancy and delivery. He supervised the writing and edited earlier drafts of the manuscript. PL treated the patient for aplastic anemia during and after pregnancy He also edited earlier drafts of the manuscript. KS did a literature study and was a major contributor to writing the manuscript. All authors read and approved the final version of the manuscript.
